# Relative Clinical and Cost Burden of Community-Acquired Pneumonia Hospitalizations in Older Adults in the United States—A Cross-Sectional Analysis

**DOI:** 10.3390/vaccines6030059

**Published:** 2018-08-31

**Authors:** Omotola Olasupo, Hong Xiao, Joshua D. Brown

**Affiliations:** Department of Pharmaceutical Outcomes and Policy, University of Florida, Gainesville, FL 32610, USA; tolaolasupo@yahoo.com (O.O.); HXiao18@cop.ufl.edu (H.X.)

**Keywords:** community-acquired pneumonia, diabetes mellitus, myocardial infarction, stroke, elderly, burden of illness

## Abstract

The relative burden of community-acquired pneumonia (CAP) in older adults (≥65 years old) compared to other serious diseases is important to prioritize preventive treatment. A retrospective analysis was conducted using the 2014 National Readmission Database to evaluate the length of stay, inpatient mortality, 30-day readmissions, and costs of CAP compared to diabetes mellitus (DM), myocardial infarction (MI), and stroke. 275,790 hospitalizations were analyzed and represented a national estimate of 616,300 hospitalizations, including 269,961 for CAP, 71,284 for DM, 126,946 for MI, and 148,109 for stroke. The mean length of stay in CAP was 5.2 days, which was higher than DM (4.6) and MI (4.3) but similar to stroke (5.6). The inpatient mortality risk was lower for DM (RR: 0.37, 95% CI: 0.29–0.46) but higher for MI (RR: 1.67, 95% CI: 1.50–1.85) and stroke (RR: 1.67, 95% CI: 1.51–1.83). The median costs for CAP ($7282) were higher compared to DM ($6217) but lower compared to MI ($14,802) and stroke ($8772). The 30-day readmission rate was 17% in CAP, which was higher compared to MI (15%) and stroke (11.5%) and lower compared to DM (20.3%). In patients with CAP, disease burden is on par with other serious diseases. CAP should be prioritized for prevention in older adults with strategies such as vaccination and smoking cessation.

## 1. Introduction

Community-acquired pneumonia (CAP) is the eighth leading cause of death and the foremost cause of death from infectious diseases in adults aged 65 years and older, therefore contributing significant morbidity and mortality in this population [1,2]. The impact of CAP is greater than in other age groups, accounting for a majority of severe cases and most of the direct medical costs [3,4]. The annual incidence of CAP in this population is four times that of younger populations. Older adults also have higher rates of hospitalization and are more likely to die as a result of CAP [4,5,6]. This disease burden in the older population is expected to further increase in the coming years, with 20% of the world’s population reaching advanced age by 2050 [3,7].

In addition to advanced age, risk factors for CAP include male sex, alcohol use, smoking, comorbidities (e.g., chronic obstructive pulmonary disease (COPD), congestive heart failure (CHF), cystic fibrosis, asthma, chronic renal disorders, hepatic conditions, diabetes mellitus, cancer, HIV infection, myeloma, surgical or “functional” asplenia), immunosuppressive conditions, and medications (e.g., inhaled corticosteroids, inhaled anticholinergics, chemotherapy, proton pump inhibitors) [8,9,10,11]. These factors further increase the risk of severe disease, which in turn increases the risk of CAP-associated deaths [11].

Diabetes mellitus (DM), myocardial infarction (MI), and stroke are also common diseases in older adults which have also been associated with significant morbidity and mortality, with tremendous attention and healthcare resources directed towards their prevention and management. These conditions receive enormous attention due to their high prevalence, modifiable risk factors, availability of proven therapies, availability of preventive strategies, potential for high disability, and substantial economic burden [12,13,14]. According to the 2010 National Hospital Discharge Survey, there were an estimated 354,000 MI hospitalizations, 663,000 stroke hospitalizations, and 198,000 DM related hospitalizations in adults aged ≥65 years old [14,15]. In comparison, there were 621,000 CAP hospitalizations in the same age group, which is similar or greater than that of MI, stroke, and DM.

While the hospital length of stay, mortality, costs, and readmissions associated with CAP have been extensively studied and compared with other serious diseases such as stroke, MI, and osteoporotic fractures, these metrics of clinical and economic disease burden have been fluctuating over the years and have not been compared specifically to other serious diseases being considered in this study [16,17,18,19]. Further, these comparative studies have relied on samples that are not representative of the entire older U.S. population. Such comparisons are informative to developing priorities for disease prevention, especially in primary care settings where clinicians must delegate short clinical visits across several disease states. The aim of this study was to estimate the length of hospital stay, inpatient mortality, costs, and 30-day readmission rates associated with CAP relative to DM, MI, and stroke in a nationally representative sample of older adults.

## 2. Materials and Methods

A retrospective cross-sectional analysis was conducted using the 2014 National Readmission Database (NRD)—a part of a family of databases developed for the Healthcare Cost and Utilization Project (HCUP) by the Agency for Healthcare Research and Quality in the United States [20]. The NRD provides information on demographics, diagnoses, procedures, comorbidities, and hospitalization outcomes in a nationally representative sample accounting for 51.2% of the U.S. population and 49.3% of all U.S. hospitalizations [20]. Unweighted, the NRD contains data from approximately 15 million discharges annually, which represents 35 million discharges once weighted to be nationally representative. Weighting accounts for demographic differences and geographic sampling so that the data can be representative of the general US population, not just of the states that are present in the database. Each individual hospitalization is deidentified and maintained as a unique entry. Use of the NRD data for this study was considered exempt by the University of Florida Institutional Review Board.

**Figure 1:**
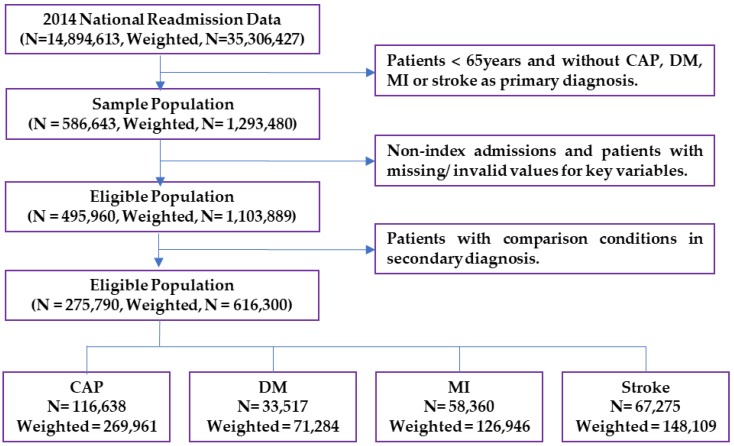
Sample selection flow chart.

Individuals aged ≥65 years old with a primary diagnosis of CAP, DM, MI, or stroke were included in the study. Cases were identified using the corresponding International Classification of Diseases, Ninth Revision, Clinical Modification (ICD-9-CM) codes listed in Appendix Table A1 [21,22]. Cases with a primary diagnosis of septicemia or respiratory failure and a secondary diagnosis of CAP were also included as CAP cases. Patients with any of the comparison conditions as a secondary diagnosis were excluded. Patients with unconfirmed values and outliers for the outcomes of interest (length of stay and charges) were excluded using quantiles for normal distribution (e.g., those less than 1% ($4273) or greater than 99% ($372,073) for the total charge and those less than 1 day or greater than 31 days for the length of stay were excluded). A flow chart of the sample selection is provided in Figure 1.

Individuals’ demographic and clinical characteristics, comorbidities, admission, and hospital-related variables were extracted from the core, and severity measures and hospital weights files of the NRD were analyzed. The number of chronic conditions, risk of mortality subclass, and severity of illness subclass were also assessed as comorbidity and risk measures [17]. A description of the data elements is provided in Appendix A.

In-hospital mortality was defined as the percentage of deaths within the index hospitalization period. Length of stay (LOS) was calculated by subtracting the admission date from the discharge date. Costs for each discharge were estimated by multiplying total charges by the all-payer inpatient cost/charge ratio, which was based on the hospital-specific all-payer inpatient cost/charge ratio (APICC) when available, or the hospital group average all-payer inpatient cost/charge ratio (GAPICC) otherwise. Total charges and costs did not include professional fees and uncovered charges. Total summed costs were also obtained for each condition. 30-day readmissions rates were also obtained in the study cohort for those who were alive at discharge.

Descriptive and summary statistics (mean, median, mode, standard deviation) were obtained for continuous variables such as age and number of chronic conditions. Frequencies were accessed for each level of categorical variables, and chi-square and Fisher’s exact tests were conducted to determine statistically significant differences in the characteristics across the four patient groups. A complete case analysis was used to address missing values for key variables. For the non-normally distributed continuous outcomes (length of stay and cost), comparisons were made using Kruskal Wallis test. The 30-day readmission rates for the conditions were also estimated.

Associations between inpatient mortality and diagnosis were examined by generalized linear models using a modified Poisson distribution to estimate the relative risk of death with 95% confidence intervals. Covariates that were shown to be significantly different across the four groups and identified as potential confounders were adjusted for in the model. CAP cases were used as the reference group in all comparisons and an a priori significance level of α = 0.05 was used in all analyses. All analyses were conducted using SAS Software 9.4 version (c) 2002–2012, SAS Institute Inc., Cary, NC, USA.

## 3. Results

A total of 275,790 hospitalizations representing a national estimate of 616,300 hospitalizations were included in this study. Of the sample, 43.8% had CAP as the primary diagnosis while 11.6% had DM, 20.6% had MI, and 24.0% had stroke as the primary diagnosis. The mean patient age was 78.8 (±8.1) years. Patients with CAP were of similar age or were older compared with patients with stroke, DM or MI. A majority of the sample were Medicare beneficiaries (91.6%), females (54.2%), living in fringe counties of metro areas of ≥1 million people (25.0%), and in areas with an estimated median household income of $40,000–50,999 (28.6%). Most patients had non-elective (94.5%) weekday admissions (74.7%). The sample population had a median of six chronic conditions, with 73.4% having hypertension as a comorbidity. Descriptive statistics for the sample population are presented in Table 1.

### 3.1. Inpatient Mortality

The inpatient mortality for CAP patients was 4.0% while those for DM, MI, and stroke patients were 0.9%, 5.4%, and 4.9% respectively (*p* < 0.0001). The overall inpatient mortality was 4.2%. In the unadjusted analysis, the risk of inpatient mortality was lower in DM (RR 0.20; 95% CI; 0.18–0.23) but higher in MI (RR 1.33; 95% CI; 1.27–1.40) and stroke patients (RR 1.19, 95% CI; 1.14–1.26) compared to CAP patients. Adjusting for observed variables, the risk of inpatient mortality remained consistently lower for DM patients (RR: 0.37, 95% CI: 0.29–0.46), higher for MI (RR: 1.67, 95%CI: 1.50–1.85), and higher for stroke patients (RR: 1.67, 95% CI: 1.51–1.83). Adjusted and unadjusted risk estimates for inpatient mortality are provided in Table 2.

An increased age was associated with an increased risk of inpatient mortality, with those aged 75–84 years having 1.35 times the risk (RR: 1.35, 95% CI: 1.23–1.49), while those aged 85–89 years had 2.07 times the risk (RR: 2.07, 95% CI: 1.86–2.30) and those aged >90 years had 2.49 times the risk of inpatient mortality (RR: 2.49, 95% CI: 2.24–2.78) compared to those aged 65–74 years.

Other factors associated with an increased risk of inpatient death include having private insurance (RR: 1.51, 95% CI: 1.32–1.74) and having other payers such as Worker’s Compensation, CHAMPUS, CHAMPVA, Title V, and other government programs (RR: 3.16, 95% CI: 2.60–3.76) as the primary expected payer compared to Medicare; living in metropolitan counties with a population of 250,000–999,999 (RR: 1.13, 95% CI: 1.02–1.25), metropolitan counties with a population of 50,000–249,999 (RR: 1.39, 95% CI: 1.23–1.58), or nonmetropolitan or micropolitan counties (RR: 1.44, 95% CI: 1.22–1.68) compared to living in central metropolitan counties with a population of ≥1 million; high disease severity and coexisting CHF (RR: 1.20, 95% CI: 1.10–1.31); coagulopathy (RR: 1.18, 95% CI: 1.05–1.32); fluid and electrolyte disorders (RR: 1.11, 95% CI: 1.03–1.19); cancer without metastasis (RR: 1.58, 95% CI: 1.35–1.85); cancer with metastasis (RR: 2.30, 95% CI: 2.00–2.64); paralysis (RR: 1.21, 95% CI: 1.04–1.41); psychoses (RR:1.26, 95% CI: 1.01–1.58); and other neurological disorders (RR: 1.16, 95% CI: 1.04–1.30).

A decreased risk of inpatient death was reported in patients with coexisting deficiency anemias (RR: 0.87, 95% CI: 0.80–0.95), depression (RR: 0.83, 95% CI: 0.73–0.94), drug abuse (RR: 0.57, 95% CI: 0.33–0.96), complicated and uncomplicated hypertension (RR: 0.88, 95% CI: 0.81–0.95), hypothyroidism (RR: 0.89, 95% CI: 0.81–0.98), obesity (RR: 0.77, 95% CI: 0.66–0.91), valvular disease (RR: 0.77, 95% CI: 0.69–0.86), weight loss (RR: 0.74, 95% CI: 0.68–0.80), and having more than seven chronic conditions compared to one to four chronic conditions (RR: 0.80, 95% CI: 0.70–0.92).

Sex, income, having Medicaid or self-pay compared to having Medicare, admission day (weekend versus weekday), admission type (elective versus non-elective), coexisting alcohol abuse, rheumatoid arthritis/collagen vascular diseases, chronic blood loss anemia, chronic pulmonary disease, liver disease, lymphoma, peripheral vascular disorders, pulmonary circulation disorders, and renal failure were not associated with an increased risk of inpatient mortality.

The hospital characteristics (size, teaching status of urban hospitals, and hospital control/ownership) were not significantly associated with inpatient mortality. However, a higher risk of inpatient mortality was recorded in hospitals in small metropolitan areas with less than 1 million residents compared to hospitals in large metropolitan areas with at least 1 million residents (RR: 1.32, 95% CI: 1.10–1.58), while a lower inpatient mortality risk was observed in hospitals in micropolitan areas compared to those in large metropolitan areas (RR: 0.67, 95% CI: 0.51–0.88). The results of the multivariate analysis for inpatient mortality are presented in Table 3.

### 3.2. Length of Hospital Stay, 30-Day Readmission Rates, and Costs

The mean (±SD) length of hospital stay among older patients hospitalized primarily for CAP was 5.2 ± 3.8 days, and this was higher compared to DM (4.6 ± 3.8 days, *p* < 0.0001) and MI patients (4.3 ± 3.6 days, *p* < 0.0001). The length of hospitalization for CAP and stroke patients (5.6 ± 5.5 days) was similar, with a median length of hospital stay of four days for both conditions. The 30-day readmission rate was 17% in CAP patients, which is higher compared to MI (15%) and stroke (11.5%) patients. DM had the highest 30-day readmission rate at 20.3%.

**Figure 2:**
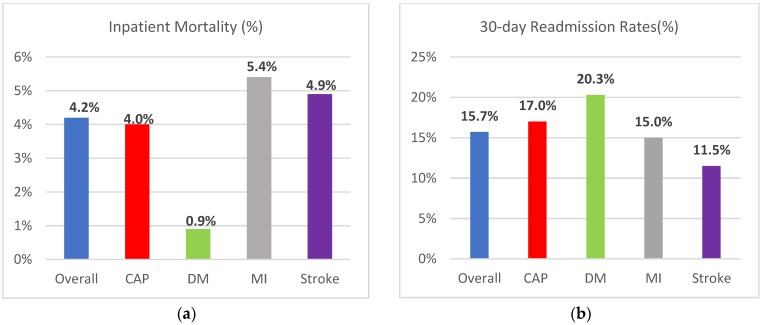

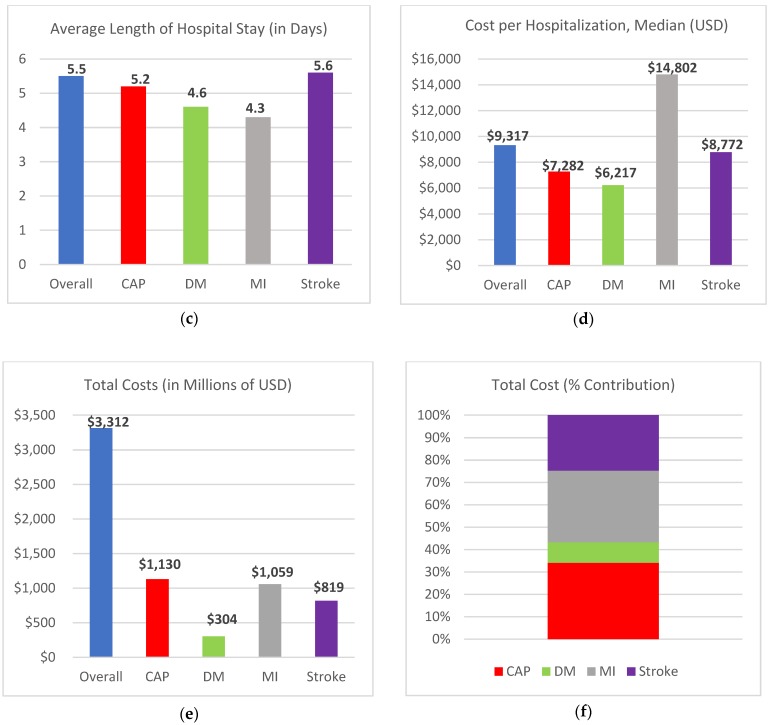
Comparison of hospitalization metrics for CAP, DM, MI, and stroke (**a**) Inpatient mortality; (**b**) 30-day readmissions rates; (**c**) length of hospital stay; (**d**) median cost per hospitalization; (**e**) total cost in millions of US dollars; (**f**) total cost in % contribution.

The average cost of each hospitalization for CAP ($9686 ± $8340) was higher than the average cost for a DM hospitalization ($9057 ± $9246), while the cost/hospitalization was higher for MI and stroke compared to CAP at $18,148 ± 13,088 and $12,188 ± 10,283 respectively. For the study year, CAP accounted for the highest total costs, with the total cost being $1130 million while DM costed $304 million, MI costed $1059 million, and stroke costed $819 million. The total hospitalization costs attributed to CAP alone exceeded the cost attributed to DM and stroke combined. Estimates for the length of hospital stay, costs, and 30-day readmissions are provided in Table 4. A comparison of hospitalization metrics for CAP, DM, MI, and stroke are also provided in Figure 2.

## 4. Discussion

This study estimated the mean length of hospital stay, inpatient mortality, cost per discharge, total costs, and 30-day readmission associated with CAP relative to DM, MI, and stroke in older adults aged ≥65 years. Compared to previous studies, the findings of this study are consistent with findings such as the LOS of ≥5 days for CAP as reported by File et al. [16], the effect of increased age on inpatient mortality as reported by Kaplan et al. [5], and the non-effect of sex on inpatient mortality risk [5]. The association between variables such as inpatient mortality, comorbid conditions, and CAP severity was also consistent with findings by Welte et al. in a European cohort [18]. Also, in a comparative burden of illness study by Brown et al. in a population of Medicare Advantage with Prescription Drug Plan (MAPD) beneficiaries, the mean LOS for CAP hospitalizations (5.2 days) was statistically longer than MI, stroke, and osteoporotic fractures (OF). The 30-day readmission rates for CAP (10%) were also higher compared to 7.7% for stroke and 7.9% for OF [14].

Our study confirms that DM, MI, and stroke are important disease states contributing to significant morbidity and mortality in individuals aged ≥65 years, thereby justifying the large focus of primary care providers and managed care organizations on these conditions, especially since there are effective preventive medications available. CAP was, however, shown to have a comparable and often higher disease burden compared to these conditions, therefore accounting for the higher risk of inpatient mortality compared to DM, more hospitalization days compared to DM and MI, higher 30-day readmission rates than MI and stroke, and higher average costs per hospitalization compared to DM.

Another major finding of this study is that of the four conditions evaluated, CAP hospitalizations were associated with the highest total cost in the study year. In this cohort, which was representative of the older adult US population, the primary payer spent $304 million on DM hospitalizations, $1059 on MI hospitalizations, and $819 on stroke hospitalizations. In contrast, $1130 million was spent on CAP hospitalizations alone, exceeding the cost attributed to DM and stroke combined. This finding was driven by roughly the hospitalization incidence for CAP being roughly double that of MI and stroke and even larger compared to DM.

To reduce this burden, the need for increased efforts in preventing CAP through the prioritization of pneumococcal vaccinations in older adults cannot be overemphasized. CAP is a highly preventable disease due to the high success rates achieved by vaccinations [23,24]. Compared to Global Health Goals and Healthy People 2020 targets for immunization and infectious diseases however, pneumococcal vaccination coverage rates remain suboptimal even in high risk populations such as older adults and adults 19–64 years with risk factors for pneumococcal disease [25].

According to the US CDC Advisory Committee on Immunization Practices, older adults aged ≥65 years are required to receive one dose of the pneumococcal conjugate vaccine (PCV13), followed by a dose of pneumococcal polysaccharide vaccine (PPSV23) at least one year later [26,27,28]. In adults aged 19–64 years with risk factors for pneumococcal disease, the recommendations for the pneumococcal vaccination include one dose of PCV13 and at least one dose of PPSV23 depending on the patient’s age and health status. Other recommendations by the Infectious Diseases Society of America/American Thoracic Society consensus guidelines for CAP prevention include smoking cessation and influenza vaccinations for high-risk groups [14,29,30].

The pneumococcal vaccination decreases the risk of invasive pneumococcal disease such as pneumococcal meningitis and bacteremia and the rate of CAP-associated hospitalizations [23,24]. Compared to preventive measures for DM, MI, and stroke, which could include lifestyle modifications, multidisciplinary teamwork across the healthcare spectrum, pharmacotherapy, patients’ involvement, and the associated yearly cost of prevention efforts for these diseases, the utilization and cost of the pneumococcal vaccination is potentially a very efficient and effective use of disease control resources in the older population, especially when compared to the overall relative burden of CAP in this population.

While it is warranted to focus on these commonly feared diseases in older adults, our study demonstrates the enormous clinical and total cost burden of CAP hospitalizations in the older population, with total costs attributed to CAP hospitalizations exceeding the costs attributed to DM and stroke combined due to the much higher incidence of CAP hospitalizations. This burden is further worsened by the fact that only 40–60% of CAP episodes lead to hospitalizations while the remainder are managed in outpatient or emergency care settings [19]. Thus, beyond the limitations mentioned below, this study has underestimated the true burden of CAP, therefore providing further incentives for preventive efforts.

### Limitations

The NRD provides a nationally representative database on all hospitalizations and readmissions while providing a very large sample of deidentified, patient-level information needed for analyses. Limitations for this data include a lack of detailed clinical data (e.g., laboratory values), assessment of medication use, or vaccination status. NRD data is cross-sectional for each hospitalization; thus, the timing of events for a hospitalization cannot be assessed. NRD does allow for a patient follow-up within a given year and state but cannot account for patients who are hospitalized in more than one state. Our findings may only be generalizable to the U.S. older adult population as underlying disease rates may differ across developed or undeveloped countries.

## 5. Conclusions

CAP is associated with a comparable clinical and economic burden relative to myocardial infarction (MI), stroke, and diabetes mellitus (DM) in older adults. Compared to DM, CAP is associated with a longer hospital stay, higher inpatient mortality, and higher costs per hospitalization but lower 30-day readmissions rates. Compared to MI, CAP is associated with a longer hospital stay and higher 30-day readmissions rates but a lower inpatient mortality and lower costs per hospitalization. Compared to stroke, CAP accounts for a similar length of hospital stay, lower inpatient mortality, and a lower cost per hospitalization but higher 30-day readmissions rates. The total costs associated with CAP are, however, the highest of the four conditions in the study year. Being a highly preventable disease due to the availability of effective vaccines, these findings on the burden of CAP highlight a need for an increased resource allocation in disease control efforts for CAP that is comparable to other serious conditions like DM, MI, and stroke in older adults.

## Figures and Tables

**Table 1:** Characteristics of the study population 65 years and older with a hospitalization discharge for community-acquired pneumonia (CAP), diabetes mellitus (DM), myocardial infarction (MI), or stroke, weighted national estimates in 2014 Nationwide Readmissions Database, United States.

Individual Characteristics	Weighted	CAP	DM	MI	Stroke
	N = 616,300	N = 269,961	N = 71,284	N = 126,946	N = 148,109
	100%	3.80%	11.60%	20.60%	24.00%
**Demographics**	**N (%)**	**N (%)**	**N (%)**	**N (%)**	**N (%)**
Age in years (Mean ± SD)	78.8 ± 8.1	79.7 ± 8.0	75.4 ± 7.5	77.6 ± 8.2	78.5 ± 7.9
Female	334,253 (54.2)	151,117 (56.0)	37, 047 (52.0)	58, 831 (46.3)	87, 258 (58.9)
**Patient Location: NCHS Urban-Rural Code (PL_NCHS) ^1^**					
Central counties ≥1 million	125,386 (20.4)	47,555 (17.6)	21,198 (29.7)	25,049 (19.7)	31,584 (21.3)
Fringe counties of ≥1 million	154,046 (25.0)	66,274 (24.5)	17,626 (24.7)	31,199 (24.6)	38,948 (26.3)
Metro areas of 250,000–999,999	132,376 (21.5)	56,686 (21.0)	14,027 (19.7)	28,527 (22.5)	33,136 (22.4)
Metro areas of 50,000–249,999	65,328 (10.6)	29,151 (10.8)	6067 (8.5)	14,117 (11.1)	15,993 (10.8)
Micropolitan counties	70,712 (11.5)	34,748 (12.9)	6511 (9.1)	14,663 (11.6)	14,789 (10.0)
Not metropolitan/Micropolitan	67,119 (10.9)	35,033 (13.0)	5708 (8.0)	13,057 (10.3)	13,320 (9.0)
**Median Household Income National Quartile for Patient ZIP Code**					
0–25th percentile	163,896 (27.0)	71,832 (26.6)	24,673 (34.6)	31,360 (24.7)	36,030 (24.3)
26–50th percentile (median)	173,612 (28.6)	78,077 (28.9)	18,734 (26.3)	36,538 (28.8)	40,263 (27.2)
51–75th percentile	143,008 (23.6)	62,303 (23.1)	14,480 (20.3)	30,627 (24.1)	35,598 (24.0)
76th–100th percentile	126,375 (20.8)	53,566 (19.8)	12,329 (17.3)	26,408 (20.8)	34,072 (23.0)
**Primary Expected Payer**					
Medicare	564,041 (91.6)	249,869 (92.6)	64,028 (89.8)	114,597 (90.3)	135,547 (91.5)
Medicaid	7393 (1.2)	2838 (1.1)	1741 (2.4)	1208 (1.0)	1605 (1.1)
Private insurance	34,370 (5.6)	13,112 (4.9)	4142 (5.8)	8789 (6.9)	8327 (5.6)
Self-pay	2309 (0.4)	794 (0.3)	370 (0.5)	493 (0.4)	652 (0.4)
No charge	134 (0.0)	43 (0.0)	22 (0.0)	37 (0.0)	32 (0.0)
Other	7491 (1.2)	3034 (1.1)	927 (1.3)	1741 (1.4)	1790 (1.2)
**Admission Day:** Weekday	460,414 (74.7)	201,862 (74.8)	55,676 (78.1)	93,639 (73.8)	109,237 (73.8)
**Admission Type:** Non-elective	581,468 (94.5)	252,374 (93.5)	64,903 (91.0)	121,077 (95.4)	143,114 (96.6)
**Comorbidities**					
AIDS	134 (0)	<11 (0)	46 (0)	38 (0)	48 (0)
Alcohol abuse	13,700 (2.2)	4741 (1.8)	1560(2.2)	2883 (2.3)	4516 (3.0)
Deficiency anemias	128,379 (20.8)	70,587 (26.1)	18,515 (26.0)	20,833 (16.4)	18,443 (12.5)
Congestive heart failure	105,396 (17.1)	71,244 (26.4)	13,556 (19.0)	622 (0.5)	19,974 (13.5)
Chronic pulmonary disease	194,822 (31.6)	132,651 (49.1)	12,670 (017.8)	26,654 (21.0)	22,847 (15.4)
Depression	70,617 (11.5)	36,623 (13.6)	7903 (11.1)	9872 (7.8)	16,219 (11.0)
Hypertension	452,538 (73.4)	180,662 (66.9)	56,654 (79.5)	92,833 (73.1)	122,389 (82.6)
Hypothyroidism	111,022 (18.0)	52,655 (19.5)	11,557 (16.2)	19,672 (15.5)	27,139 (18.3)
Liver disease	8409 (1.4)	3932 (1.5)	2041 (2.9)	1342 (1.1)	1094 (0.1)
Fluid and electrolyte disorders	199,800 (32.4)	105,284 (39.0)	30,861 (43.3)	29,617 (2.3)	34,039 (23.0)
Other neurological disorders	57,590 (9.3)	38,231 (14.2)	8914 (12.5)	9989 (7.9)	456 (0.3)
Obesity	42,866 (7.0)	14,900 (5.5)	10,135 (14.2)	10,061 (7.9)	7770 (5.2)
Peripheral vascular disorders	59,707 (9.7)	20,041 (7.4)	11,567 (16.2)	15,551 (12.3)	12,548 (8.5)
Pulmonary circulation disorders	28,529 (4.6)	19,370 (7.2)	2165 (3.0)	142 (0.1)	19,370 (13.1)
Renal failure	116,945 (19.0)	50,083 (18.6)	22,256 (31.2)	25,040 (19.7)	19,566 (13.2)
Solid tumor without metastasis	20,093 (3.3)	12,722 (4.7)	1775 (2.5)	2444 (1.2)	3151 (2.1)
Metastatic cancer	15,903 (2.6)	10,568 (3.9)	1331 (1.9)	1522 (1.2)	2483 (1.7)
No. of chronic conditions (Mean ± SD)	6.6 ± 2.9	6.0 ± 2.9	6.9 ± 2.9	7.1 ± 2.8	7.0 ± 2.7
Median	6	6	7	7	7
**Hospital Characteristics**					
Bed size of hospital: Large	319,657 (51.9)	125,093 (43.6)	37,579 (52.7)	74,603 (58.8)	82,382 (55.6)
**Control/Ownership of Hospital:**					
Government, nonfederal	73,390 (11.9)	35,895 (13.3)	8533 (12.0)	11,978 (9.4)	16,985 (11.5)
Private, not-profit	448,313 (72.7)	192,915 (71.5)	49,736 (69.8)	94,960 (74.8)	110,702 (74.7)
Private, invest-own	94,597 (15.3)	41,152 (15.2)	13,015 (18.3)	20,009 (15.8)	20,421 (13.8)

* For all covariates, there were significant differences among the four comparison groups (*p* < 0.0001). ^1^ PL_NCHS is a six-category urban–rural classification scheme for U.S. counties developed by the National Center for Health Statistics (NCHS) for use in health care research. The classification emphasizes urban distinctions and is unique in differentiating between central and fringe counties of large metropolitan areas. Smaller metropolitan counties are subdivided by population. Nonmetropolitan counties are divided simply into micropolitan and non-core categories. An additional description of the database and data elements is provided in Appendix A.

**Table 2:** Inpatient mortality in hospitalized older adults with CAP compared with DM, MI, and stroke.

Hospitalization	Total Sample	Died during Hospitalization	Unadjusted RR	Adjusted RR
	N (%)	N (%)		
CAP	269,961 (43.8)	10,807 (4.0)	Ref	Ref
DM	71,284 (11.6)	606 (0.9)	0.20 (0.18–0.23)	0.37 (0.29–0.46)
MI	126,946 (20.6)	6841 (5.4)	1.33 (1.27–1.40)	1.67 (1.50–1.85)
Stroke	148,109 (24.0)	7309 (4.9)	1.19 (1.14–1.26)	1.67 (1.51–1.83)
Total	616,300 (100%)	25,563 (4.2)	-	-

**Table 3:** Multivariable regression results showing risk factors for inpatient mortality among the total cohort controlling for primary diagnosis.

Factors	Risk Ratio	95% CI		*p*-Value
**Age in Years at Admission**				
75–84 vs. 65–74	1.35	1.23	1.49	<0.0001
85–89 vs. 65–74	2.07	1.86	2.30	<0.0001
90+ vs. 65–74	2.49	2.24	2.78	<0.0001
**Sex:** Male vs. Female	0.95	0.89	1.02	0.17
**Patient Location: NCHS Urban–Rural Code**				
Central counties of metro areas of ≥1 million population (Ref)	-	-	-	-
Fringe counties of metro areas of ≥1 million population	0.98	0.88	1.09	0.69
Counties in metro areas of 250,000–999,999 population	1.13	1.02	1.25	0.02
Counties in metro areas of 50,000–249,999 population	1.39	1.23	1.58	<0.0001
Micropolitan counties	1.05	0.88	1.24	0.62
Not metropolitan or micropolitan counties	1.44	1.22	1.68	<0.0001
**Median Household Income National Quartile for Patient’s ZIP Code**				
0–25th percentile (Ref)	-	-	-	-
26th to 50th percentile (median)	0.96	0.88	1.06	0.45
51st to 75th percentile	1.02	0.92	1.13	0.69
76th to 100th percentile	1.09	0.98	1.21	0.13
**Primary Expected Payer**				
Medicaid vs. Medicare	1.08	0.76	1.52	0.67
Private insurance vs. Medicare	1.51	1.32	1.74	<0.0001
Self-pay vs. Medicare	1.29	0.81	2.05	0.29
No charge vs. Medicare	0.00	0.00	N/A	1.00
Others vs. Medicare	3.12	2.60	3.76	<0.0001
**Admission Day:** Admitted Weekday vs. Weekend	1.03	0.96	1.12	0.39
**Admission Type:** Non-elective vs. Elective admission	1.11	0.90	1.36	0.33
**All Patient Refined DRG: Severity of Illness Subclass**				
Minor loss of function (Ref)	-	-	-	-
Moderate loss of function	2.02	1.49	2.73	<0.0001
Major loss of function	9.57	7.14	12.84	<0.0001
Extreme loss of function	48.57	36.15	65.26	<0.0001
**Comorbidities**				
AIDS	0.00	0.00	N/A	1.00
Alcohol abuse	1.24	0.99	1.55	0.06
Deficiency anemias	0.87	0.80	0.95	0.00
Rheumatoid arthritis/collagen vascular diseases	1.07	0.89	1.29	0.45
Chronic blood loss anemia	0.73	0.49	1.10	0.14
Congestive heart failure	1.20	1.10	1.31	<0.0001
Chronic pulmonary disease	0.93	0.86	1.01	0.08
Coagulopathy	1.18	1.05	1.32	0.01
Depression	0.83	0.73	0.94	0.00
Drug abuse	0.57	0.33	0.96	0.04
Hypertension (combine uncomplicated and complicated)	0.88	0.81	0.95	0.00
Hypothyroidism	0.89	0.81	0.98	0.02
Liver disease	1.21	0.94	1.56	0.14
Lymphoma	0.77	0.58	1.02	0.07
Fluid and electrolyte disorders	1.11	1.03	1.19	0.00
Other neurological disorders	1.16	1.04	1.30	0.01
Obesity	0.77	0.66	0.91	0.00
Paralysis	1.21	1.04	1.41	0.01
Peripheral vascular disorders	0.97	0.87	1.09	0.65
Psychoses	1.26	1.01	1.58	0.04
Pulmonary circulation disorders	0.94	0.82	1.08	0.41
Renal failure	0.96	0.88	1.05	0.34
Solid tumor without metastasis	1.58	1.35	1.85	<0.0001
Metastatic cancer	2.30	2.00	2.64	<0.0001
Valvular disease	0.77	0.69	0.86	<0.0001
Weight loss	0.74	0.68	0.80	<0.0001
**Number of Chronic Conditions**				
1–4 (Ref)	-	-	-	-
5–6	0.90	0.81	1.00	0.06
7–8	0.84	0.75	0.95	0.00
>8	0.80	0.70	0.92	0.00
**Hospital Characteristics**				
**Bed size of hospital:** Medium vs. Small	1.06	0.94	1.18	0.35
Large vs. Small	1.00	0.90	1.11	0.99
**Hospital urban–rural designation**				
Large metropolitan areas with at least 1 million residents (Ref)	-	-	-	-
Small metropolitan areas with less than 1 million residents	1.32	1.10	1.58	0.0025
Micropolitan areas	0.67	0.51	0.88	0.0044
Not metropolitan or micropolitan (non-urban residual)	1.00	1.00	1.00	N/A
**Teaching status of urban hospitals:**				
Metropolitan teaching vs. metropolitan non-teaching	0.91	0.84	0.99	0.02
**Control/ownership of hospital:**				
Private, not-profit vs. government, nonfederal	1.01	0.92	1.12	0.80
Private, invest-own vs. government, nonfederal	1.14	1.00	1.30	0.04

**Table 4:** Length of hospital stay, costs, and 30-day readmission rates among older adults hospitalized for targeted conditions.

Hospitalization Metric	Overall Sample	CAP	DM	MI	Stroke	*p*-Value
LOS (days)	5.5 ± 4.7	5.2 ± 3.8	4.6 ± 3.8	4.3 ± 3.6	5.6 ± 5.5	-
LOS (Median)	4	4	3	3	4	<0.0001
Total charges	$51,612 ± 52,478	$35,480 ± 36,149	$35,341 ± 38,580	$72,077 ± 60,105	$46,785 ± 44,219	-
Total charges (Median)	$33,637	$24,309	$23,222	$56,016	$32,458	<0.0001
Total Cost	$13,425 ± 12,366	$9686 ± 8340	$9057 ± 9246	$18,148 ± 13,088	$12,188 ± 10,283	-
Total Cost (Median)	$9317	$7282	$6217	$14,802	$8772	<0.0001
Total summed costs (Millions)	$3312	$1130	$304	$1059	$819	<0.0001
30-day readmission rates	15.7%	17.0%	20.3%	15.0%	11.5%	<0.0001

LOS = Length of stay.

## Appendix A

**Table A1:** Disease state ICD-9 Coding.

Disease State	ICD-9 Codes
Community-Acquired Pneumonia (CAP)	(1) 480.x–486.x, 487.0 Pneumonia as a principal diagnosis; OR (2) 518.8x Respiratory Failure or 038.xx Septicemia as a principal diagnosis with Pneumonia as a secondary diagnosis on the same claim line
Myocardial Infarction	410.xx, 412.xx
Stroke	433.x1, 434.x1, 436.xx
Diabetes Mellitus (with or without complications)	250.0x–250.9x
